# The Value of Different Single or Combined Indexes of the Captopril Challenge Test in the Diagnosis of Primary Aldosteronism

**DOI:** 10.3389/fendo.2021.689618

**Published:** 2021-06-17

**Authors:** Qiao Xiang, Tao Chen, Kai Yu, Yuanmei Li, Qianrui Li, Haoming Tian, Yan Ren

**Affiliations:** ^1^ Department of Endocrinology and Metabolism, Adrenal Center, Sichuan University West China Hospital, Chengdu, China; ^2^ Department of Endocrinology and Metabolism, Suining Central Hospital, Suining, China; ^3^ Department of Nuclear Medicine, Sichuan University West China Hospital, Chengdu, China; ^4^ Chinese Evidence-Based Medicine Centre and CREAT Group, State Key Laboratory of Biotherapy, West China Hospital, Sichuan University and Collaborative Innovation Centre, Chengdu, China

**Keywords:** captopril challenge test, primary aldosteronism, essential hypertension, diagnosis, aldosterone to renin ratio

## Abstract

**Objective:**

The result interpretation of the captopril challenge test (CCT) for the diagnosis of primary aldosteronism (PA) is not standardized. Superiorities of different indexes in the CCT have not been fully investigated. We aimed to comprehensively evaluate the value and influence factors of different CCT-associated indexes in the diagnosis of PA.

**Methods:**

We enrolled 312, 85, 179 and 97 patients in the groups of PA, essential hypertension (EH), unilateral PA (UPA) and bilateral PA (BPA), respectively. For each single index investigated, we computed diagnostic estimates including the area under the receiver operating characteristic curve (AUC). We performed pre-specified subgroup analyses to explore influence factors. We assessed the diagnostic value of combined indexes in binary logistic regression models.

**Results:**

Post-CCT aldosterone to renin ratio (ARR) (AUC = 0.8771) and plasma aldosterone concentration (PAC) (AUC = 0.8769) showed high value in distinguishing PA from EH, and their combination (AUC = 0.937) was even superior to either alone. The diagnostic efficacy was moderately high for post-CCT aldosterone to angiotensin II ratio (AA2R) (AUC = 0.834) or plasma renin activity (PRA) (AUC = 0.795) but low for the suppression percentage of PAC (AUC = 0.679). Post-CCT PAC had a significantly higher AUC in the UPA than BPA subgroup (AUC = 0.914 *vs* 0.827, P<0.05).

**Conclusion:**

We can take post-CCT ARR and PAC altogether into account to distinguish PA from EH, while caution should be taken to interpret CCT results with the suppression percentage of PAC. Post-CCT PAC may perform better to identify the unilateral than bilateral form of PA.

## Introduction

Primary aldosteronism (PA) is caused by idiopathic hyperaldosteronism (IHA) or an aldosterone-producing adenoma (APA), leading to inappropriately high and partly autonomous aldosterone secretion (1–3). PA is one of the leading causes of secondary hypertension, with a prevalence of 5% to 10% in the hypertensive population and up to 17% to 23% in patients with resistant hypertension (4–7). Compared with blood-pressure-matched patients with essential hypertension (EH), PA patients have higher cardiovascular morbidity and mortality (8–10), which makes the early diagnosis of and intervention in PA very important.

The aldosterone to renin ratio (ARR), which is the plasma aldosterone concentration (PAC) divided by plasma renin activity (PRA), is recommended by clinical practice guidelines for PA screening (2, 11, 12). People who have positive screening results, which mean inappropriate elevations of the ARR exceeding a certain threshold, need additional tests for confirmation. The captopril challenge test (CCT), first proposed by Lyons (13) in 1983, is now one of the four confirmatory tests recommended by the American, Japanese, or Chinese guidelines or consensus. Compared with the saline infusion test (SIT), oral sodium loading test, and fludrocortisone suppression test, the CCT is favorable due to improved security and feasibility, a lower incidence of sharp fluctuations in blood pressure, less time and expense, and not being affected by daily sodium intake (2, 11, 12).

However, the interpretation of CCT results has not yet been standardized. The present recommendation in the guidelines and the most widely adopted option in clinical practice is to interpret results with the post-CCT suppression percentage of PAC (2, 12). The post-CCT absolute value of PAC is recommended by the Japanese guidelines (11) and adopted in other studies as the discriminatory standard (13, 14). But for other indexes, including the post-CCT absolute value or percentage change of PRA, angiotensin II (AT II), ARR, aldosterone to AT II ratio (AA2R), evidence on their diagnostic value is limited or disputable. Besides post-CCT PAC, the less commonly used index—post-CCT ARR—is also recommended by the Japanese guidelines (11), but its diagnostic value and optimal cutoff values are controversial in different studies (7, 13, 15–23). AA2R was proven by a previous study to be powerful and cost-effective in the diagnosis of PA (24), although a Chinese study by Li YM et al. did not recommend its use in PA screening (unpublished data), and the diagnostic value of CCT-associated AA2R remains unknown. Moreover, different combinations of the above CCT-associated indexes for the diagnosis of PA have been rarely investigated. And it is not clear whether the diagnostic efficacy of the CCT is affected by different postures (upright or supine position) during blood sampling, serum potassium status, and findings on adrenal imaging.

Meanwhile, current guidelines and expert consensus suggest that PA be screened and diagnosed in the hypertensive population with risk factors of PA (2, 11, 12), while data on changes in aldosterone levels of EH patients are insufficient for result interpretation. Several Chinese studies even showed similar suppression percentages of PAC after the CCT between PA and EH (25–27), challenging its diagnostic value in distinguishing PA from EH. In addition, the American guidelines further interprets that the response of aldosterone after the CCT may differ between different subtypes of PA, including APA and IHA, and a certain degree of aldosterone suppression can only be seen in a minority of patients with IHA (2).

Hence, we retrospectively and comprehensively evaluated the value of different single or combined indexes of the CCT in the diagnosis of PA. We also explored the potential influence factors of the CCT.

## Methods

### Ethics

Data extraction was approved by the Ethical Committee of Sichuan University West China Hospital and adhered to the principles of the Declaration of Helsinki.

### Study Subjects

Based on the electronic medical record (EMR) system of Sichuan University West China hospital, we extracted data of individuals who were discharged with diagnoses of PA (from the departments of endocrinology, cardiology, and urology) or EH (from the department of endocrinology) on their front sheets of medical records from January 1, 2009, to December 31, 2019. We filled in the missing data by manually searching in the EMR system. After data supplementation, we excluded individuals who still lacked relevant data on results of the CCT, had severely impaired renal function as indicated by estimated glomerular filtration rate (eGFR) < 30 ml/min/1.73 m^2^), or did not conform to requirements of medication adjustment for the screening test (see the section of *Test Methods* for more details).

### Inclusion and Exclusion Criteria

To make sure the validity of discharge diagnoses, we further validated the individuals’ diagnoses and developed the criteria for inclusion and exclusion.

Patients were enrolled in the PA group if they met at least one of the two following criteria: 1) they had positive results of both the upright screening test (ARR>30 (ng/dl)/(ng/ml/h); or ARR>20 (ng/dl)/(ng/ml/h) and PRA<1 ng/ml/h; or ARR>20 (ng/dl)/(ng/ml/h) and PAC>15 ng/dl) and the saline infusion test (SIT) (post-SIT PAC>10 ng/dl); 2) they had hypokalemia along with PAC>20 ng/dl and low renin levels below the limit of detection in the upright screening test. Exclusion criteria for PA were diseases leading to secondary aldosteronism, such as renal artery stenosis, juxtaglomerular cell tumor.

For patients who were confirmed to have PA, noncosyntropin-stimulated adrenal vein sampling (AVS) with sequential cannulation was performed, and successful catheterization was defined as the selective index (the adrenal to peripheral vein cortisol ratio) ≥2. They were further classified into the unilateral PA (UPA) group if they showed lateralization of aldosterone secretion at AVS, indicated by the lateralization index (the aldosterone to cortisol ratio between the dominant and nondominant adrenal gland) ≥2; those who had a lateralization index <2 were classified into the bilateral PA (BPA) group (28).

Patients were enrolled in the EH group if: 1) they had hypertension based on the diagnostic standard suggested in the 2018 Chinese guidelines for the management of hypertension (29): systolic blood pressure (SBP) ≥ 140 mm Hg and/or diastolic blood pressure (DBP) ≥ 90 mm Hg under no antihypertensive treatment in clinic (1 mm Hg = 0.133 kPa); and 2) their standard workup ruled out PA and other comorbidities, which could lead to secondary hypertension, such as renal artery stenosis, chronic kidney disease, pheochromocytoma, Cushing’s syndrome, congenital adrenocortical hyperplasia, hyperthyroidism, hypothyroidism, and juxtaglomerular cell tumor.

### Data Collection

We extracted the following data from the EMR system (data regarding ARR, AA2R, suppression or increasing percentage were obtained by calculation).

Clinical data: gender, age, body mass index (BMI), SBP, and DBP;
Laboratory data: baseline PAC, PRA, AT II, ARR, AA2R both in the upright and supine position; serum potassium and the corresponding serum potassium status (“hypokalemia” or “non-hypokalemia”); eGFR, serum sodium and serum chlorine; PAC, PRA, AT II, ARR, AA2R before and after the CCT (pre- and post-PAC, pre- and post-PRA, pre- and post-AT II, pre- and post-ARR, pre- and post-AA2R); suppression percentage of PAC, AT II, ARR, AA2R in the CCT (PAC SP, AT II SP, ARR SP, AA2R SP); increasing percentage of PRA in the CCT (PRA IP);
Other data: patient posture during blood sampling of the CCT; findings on the adrenal computed tomography (CT) scan (“normal imaging” or “abnormal imaging”); aldosterone and cortisol levels measured in AVS.

For patients with more than one record of these data, baseline PAC, PRA, AT II, ARR, AA2R each referred to the corresponding value in the latest hormonal screening test during hospitalization; SBP and DBP referred to the mean value during hospitalization; serum potassium referred to the record concomitant with the CCT in the EMR system, and hypokalemia was defined as serum potassium <3.5 mmol/L; eGFR, serum sodium, and serum chlorine each referred to the corresponding result in the initial blood biochemical test on admission. Abnormal imaging was defined as a sign of CT-indicated lesions (e.g., adenoma or hyperplasia).

### Test Methods

Antihypertensive medications were discontinued before the standard workup, including angiotensin-converting enzyme inhibitors (ACEIs), angiotensin II receptor blockers (ARBs), β-blockers, diuretics for at least 2 weeks, and spironolactone for at least 6 weeks, but non-dihydropyridine calcium channel blockers (NDHP-CCBs) and α-blockers could be administered when marked high blood pressure required controlling. Other medications potentially interfering with the renin-angiotensin-aldosterone system (RAAS) were also discontinued, including licorice, steroids, non-steroidal anti-inflammatory drugs, sex hormones. Patients consumed a usual diet with no restrictions on sodium intake during the diagnostic procedures.

As recommended by the guidelines (2), blood sampling for the upright screening test was performed after at least 2 h of standing in the morning. The CCT was conducted in the supine or upright position with a captopril dose of 50 mg and a 120-min interval between captopril administration and blood sampling. The SIT was conducted after resting for 1 h in the supine position with an intravenous drip infusion of 2 L of 0.9% saline administered to patients within 4 h. Study patients underwent enhanced CT scanning of the adrenal gland, and experienced radiologists from the radiology department of our hospital independently assessed images in a blinded way.

Levels of PAC, PRA, and AT II were measured by radioimmunoassay using a commercial kit (Jiuding Biological Technology Ltd., Tian Jin, China) with the intra- and inter-assay coefficients of variation being 7.3% and 9.6%, respectively. The limit of detection of PRA was 0.1 ng/ml/h.

### Statistical Analysis

Continuous variables in normal distribution were presented as mean and standard deviation (SD), while those in skewness distribution were presented as median and interquartile range (Q1–Q3). Continuous variables were compared using the Student’s t test when the data were normally distributed or Kruskal-Wallis H test when the data were non-normally distributed. Categorical variables were presented as number and percentage (%), and they were compared using the Chi-square test or Fisher’s exact test.

We constructed the receiver operating characteristic (ROC) curve and computed the relevant area under the curve (AUC) to assess the diagnostic value of the following single indexes in the diagnosis of PA: post-PAC, post-PRA, post-AT II, post-ARR, post-AA2R, PAC SP, AT II SP, ARR SP, AA2R SP, and PRA IP. We determined the optimal cutoff value of each index according to the Youden’s index and computed the corresponding sensitivity (Sen), specificity (Spe) at that threshold. We made pairwise comparison of the top 6 single indexes with the largest AUC as proposed by DeLong et al. (30). We further conducted subgroup analyses on the diagnostic value of CCT-associated single indexes stratified by the PA subtype (UPA or BPA subgroup); posture during blood sampling (“upright CCT” or “supine CCT” subgroup); serum potassium status (“non-hypokalemia” or “hypokalemia” subgroup) and finding on adrenal CT (“normal imaging” or “abnormal imaging” subgroup). For the top 5 indexes with the largest AUC, we combined any two of them to form new combined indexes. To assess the diagnostic value of combined indexes, we included different combinations of indexes in the binary logistic regression models and applied the coefficients obtained from the models to our study patients to calculate predicted probabilities by the equation: ŷ = 1/[1 + exp. (−xβ)].

All statistical analyses were performed using SPSS version 20.0, MedCalc version 8.0 or R packages (http://www.R-project.org; TeRFoundation) of EmpowerStats software. P values ≤ 0.05 were considered statistically significant.

## Results

### Patient Selection

We extracted data of 1059 patients in total who were discharged with diagnoses of PA or EH on their front sheets of medical records (from January 1, 2009, to December 31, 2019). Of them, 662 patients were excluded due to insufficient data on either the screening test or the CCT even after manual supplementation for missing data, severely impaired renal function, or non-conformity with requirements of medication adjustment. From the remaining 397 patients, 312 and 85 patients were finally enrolled in the PA and EH groups, respectively. Among the 312 patients with PA, 179 were enrolled in the UPA group, while 97 of them were enrolled in the BPA group. We could not determine the subtype of the remaining 36 patients with PA because they failed in AVS cannulation or were unwilling to undergo AVS procedures.

236 patients from the PA group and 60 patients from the EH group conducted the CCT in the upright position during blood sampling, while 76 patients from the PA group and 25 patients from the EH group conducted the CCT in the supine position.

### Data Comparison Between Groups

#### The PA *vs.* EH Group

Baseline PAC, ARR, AA2R in the upright position, baseline PAC, ARR, AA2R in the supine position, DBP and serum sodium were significantly higher in the PA group than the EH group (P<0.05). Baseline PRA, AT II in the upright position, baseline PRA in the supine position, serum chlorine, and serum potassium were significantly higher in the EH group than the PA group (P<0.05). The incidence of hypokalemia was significantly higher in the PA group [45.19% (141/312)] than the EH group [22.35% (19/85)], and the proportion of patients with abnormal findings on adrenal CT was also significantly higher in the PA group [86.22% (269/312)] than the EH group [32.94% (28/85)] (P<0.05). No significant differences were found in gender, age, BMI, SBP, eGFR, and baseline AT II in the supine position between the two groups (P>0.05) (**Table 1**).

**Table 1:** Comparison of baseline data between groups (PA *vs* EH and UPA *vs* BPA).

	PA (N=312)	EH (N=85)	P value (PA *vs* EH)	UPA (N=179)	BPA (N=97)	P value (UPA *vs* BPA)
Age (yr)	48.64 (11.64)	50.22 (13.02)	0.28	46.40 (11.29)	50.91 (11.58)	0.002
Gender: Female N (%)	183 (58.65%)	49 (57.65%)	0.867	103 (57.54%)	58 (59.79%)	0.717
Adrenal imaging: abnormal N (%)	269 (86.22%)	28 (32.94%)	<0.001	175 (97.77%)	71 (73.20%)	<0.001
Serum potassium status: hypokalemia N (%)	141 (45.19%)	19 (22.35%)	<0.001	161 (89.94%)	67 (69.07%)	<0.001
BMI (kg/m^2^)	24.52 (3.32)	24.67 (3.26)	0.713	24.22 (3.46)	25.09 (3.05)	0.043
SBP (mm Hg)	150.00 (137.50–161.00)	146.60 (15.11)	0.144	152.00 (140.75–165.00)	145.00 (136.00–158.00)	0.017
DBP (mm Hg)	94.64 (12.88)	91.02 (12.66)	0.022	97.05 (12.67)	91.96 (12.84)	0.002
eGFR (ml/min/1.73 m^2^)	102.85 (93.10–112.48)	100.09 (16.27)	0.299	103.54 (95.78–115.06)	101.52 (89.01–110.31)	0.076
Serum potassium (mmol/L)	2.80 (2.31–3.34)	3.54 (3.25–3.74)	<0.001	2.60 (0.62)	3.20 (2.63–3.60)	<0.001
Serum sodium (mmol/L)	143.61 (2.49)	142.53 (2.46)	<0.001	143.98 (2.49)	143.22 (2.43)	0.017
Serum Chlorine (mmol/L)	103.45 (101.60–105.00)	104.38 (3.04)	0.003	103.40 (101.30–105.00)	103.49 (2.68)	0.311
Upright PAC (ng/dl)	27.09 (20.88–36.68)	20.28 (15.73–26.37)	<0.001	29.65 (21.98–39.68)	26.32 (20.48–33.15)	0.05
Upright PRA (ng/ml/h)	0.29 (0.10- 0.65)	1.72 (0.96–12.00)	<0.001	0.20 (0.10–0.54)	0.35 (0.12–0.80)	0.023
Upright AT II (ng/L)	60.07 (53.03–68.84)	67.73 (56.72–74.77)	0.001	57.59 (51.61–65.69)	64.07 (56.35–73.49)	<0.001
Upright ARR (ng/dl)/(ng/ml/h)	96.34 (39.47–245.33)	9.95 (2.20–18.10)	<0.001	142.36 (46.17–300.81)	76.76 (34.76–173.78)	0.005
Upright AA2R (ng/dl)/(ng/L)	0.46 (0.34–0.66)	0.34 (0.25–0.43)	<0.001	0.54 (0.37–0.72)	0.39 (0.31–0.50)	<0.001
Supine PAC (ng/dl)	25.94 (18.63–36.44)	15.98 (12.77–20.01)	<0.001	31.63 (20.59–42.09)	21.58 (17.75–27.90)	<0.001
Supine PRA (ng/ml/h)	0.1 (0.10–0.17)	0.4 (0.15–0.92)	<0.001	0.1 (0.10–0.16)	0.1 (0.10–0.18)	0.943
Supine AT II (ng/L)	59.38 (51.41–67.07)	59.55 (49.24–67.72)	0.863	58.73 (49.77–66.47)	61.39 (55.10–67.84)	0.094
Supine ARR (ng/dl)/(ng/ml/h)	213.55 (120.75–328.75)	39.29 (20.51–96.73)	<0.001	253.25 (135.56–381.69)	179.25 (105.33–241.90)	<0.001
Supine AA2R (ng/dl)/(ng/L)	0.45 (0.30–0.66)	0.30 (0.23–0.36)	<0.001	0.56 (0.33–0.79)	0.34 (0.28–0.47)	<0.001

Data were presented as mean (standard deviation), median (interquartile range: Q1-Q3) or N (%) as described before. PA, primary aldosteronism; EH, essential hypertension; UPA, unilateral primary aldosteronism; BPA, bilateral primary aldosteronism; BMI, body mass index; SBP, systolic blood pressure; DBP, diastolic blood pressure; eGFR, estimated glomerular filtration rate; PAC, plasma aldosterone concentration; PRA, plasma renin activity; AT II, angiotensin II; ARR, aldosterone to renin ratio; AA2R, aldosterone to angiotensin II ratio.

The PA group had significantly higher pre-PAC, post-PAC, pre-ARR, post-ARR, pre-AA2R, post-AA2R, AT II SP but significantly lower pre-PRA, post-PRA, post-AT II, PAC SP, PRA IP, ARR SP, AA2R SP than the EH group (P<0.05). Based on the medians of PAC SP, aldosterone was suppressed by 14% and 26% after the CCT in the PA and EH groups, respectively, but both below a suppression percentage of 30%. No significant differences were found in pre-AT II (P>0.05) (**Table 2**).

**Table 2:** Comparison of CCT-associated data between groups (PA vs EH and UPA vs BPA).

	PA (N=312)	EH (N=85)	P value (PA *vs* EH)	UPA (N=179)	BPA (N=97)	P value(UPA *vs* BPA)
Pre-PAC (ng/dl)	27.91 (22.20–38.88)	19.75 (15.95–24.35)	<0.001	32.55 (25.01–42.07)	24.17 (19.76–32.52)	<0.001
Post-PAC (ng/dl)	23.48 (18.70–33.20)	14.42 (11.62–17.22)	<0.001	27.59 (21.90–36.15)	21.08 (16.70–28.29)	<0.001
PAC SP	0.14 (0.03–0.26)	0.26 (0.15–0.39)	<0.001	0.16 (0.03–0.26)	0.11 (0.19)	0.263
Pre-PRA (ng/ml/h)	0.10 (0.10–0.31)	0.76 (0.28–2.02)	<0.001	0.10 (0.10–0.35)	0.10 (0.10–0.30)	0.824
Post-PRA (ng/ml/h)	0.20 (0.10–0.60)	1.51 (0.63–6.26)	<0.001	0.18 (0.10–0.60)	0.24 (0.10–0.57)	0.827
PRA IP	0.15 (0.00–1.28)	0.52 (0.07–1.96)	0.011	0.09 (0.00–1.20)	0.20 (0.00–1.54)	0.231
Pre-AT II (ng/L)	58.90 (51.24–67.04)	60.25 (50.32–69.39)	0.819	58.53 (51.03–66.45)	59.76 (53.40–68.96)	0.204
Post-AT II (ng/L)	57.38 (50.83–66.82)	62.00 (50.77–70.44)	0.036	56.93 (50.02–66.68)	58.53 (53.87–68.33)	0.07
AT II SP	0.02 (−0.06 to 0.11)	−0.04 (−0.18 to 0.06)	<0.001	0.02 (−0.08 to 0.12)	0.02 (−0.06 to 0.08)	0.702
Pre-ARR (ng/dl)/(ng/ml/h)	198.70 (97.22–312.55)	23.37 (10.69–49.73)	<0.001	219.27 (102.25–351.20)	176.60 (87.32–259.91)	0.027
Post-ARR (ng/dl)/(ng/ml/h)	110.71 (38.59–218.90)	9.38 (2.85- 21.38)	<0.001	127.41 (45.48–263.76)	94.88 (35.03–183.10)	0.043
ARR SP	0.30 (0.11–0.60)	0.55 (0.33–0.75)	<0.001	0.28 (0.08–0.58)	0.36 (0.13–0.65)	0.42
Pre-AA2R (ng/dl)/(ng/L)	0.50 (0.35–0.74)	0.35 (0.28–0.42)	<0.001	0.58 (0.40–0.82)	0.41 (0.30–0.53)	<0.001
Post-AA2R (ng/dl)/(ng/L)	0.44 (0.31–0.63)	0.23 (0.18–0.33)	<0.001	0.52 (0.35–0.69)	0.36 (0.26–0.49)	<0.001
AA2R SP	0.12 (−0.06 to 0.25)	0.31 (0.16–0.42)	<0.001	0.13 (−0.02 to 0.26)	0.10 (−0.08 to 0.26)	0.554

Data were presented as mean (standard deviation), median (interquartile range: Q1-Q3) or N (%) as described before. PA, primary aldosteronism; EH, essential hypertension; UPA, unilateral primary aldosteronism; BPA, bilateral primary aldosteronism; PAC, plasma aldosterone concentration; PRA, plasma renin activity; AT II, angiotensin II; ARR, aldosterone to renin ratio; AA2R, aldosterone to angiotensin II ratio; Pre-PAC/PRA/AT II/ARR/AA2R, PAC/PRA/AT II/ARR/AA2R before the CCT; Post-PAC/PRA/AT II/ARR/AA2R, PAC/PRA/AT II/ARR/AA2R after the CCT; PAC/ATII/ARR/AA2R SP, suppression percentage of PAC/ATII/ARR/AA2R in the CCT; PRA IP, increasing percentage of PRA in the CCT.

#### The UPA *vs.* BPA Group

Baseline PAC, ARR, AA2R in the upright position, baseline PAC, ARR, AA2R in the supine position, SBP, DBP, and serum sodium were significantly higher in the UPA group than the BPA group (P ≤ 0.05). Baseline PRA, AT II in the upright position, age, BMI, and serum potassium were significantly higher in the BPA group than the UPA group (P<0.05). The incidence of hypokalemia was significantly higher in the UPA group [89.94% (161/179)] than the BPA group [69.07% (67/97)], and the proportion of patients with abnormal findings on adrenal CT was also significantly higher in the UPA group [97.77% (175/179)] than the BPA group [73.20% (71/97)] (P < 0.05). No significant differences were found in baseline PRA, AT II in the supine position, gender, eGFR, and serum chlorine between the two groups (P>0.05) (**Table 1**).

The UPA group had significantly higher pre-PAC, post-PAC, pre-ARR, post-ARR, pre-AA2R, and post-AA2R than the BPA group (P<0.05). Based on the medians of PAC SP, aldosterone was suppressed more in the UPA group (by 16%) than the BPA group (by 11%) after the CCT, although the difference was not significant (P>0.05). Besides, no significant differences were found in pre-PRA, post-PRA, pre-AT II, post-AT II, PRA IP, AT II SP, ARR SP, and AA2R SP (P>0.05) (**Table 2**).

### Assessment on the Value Of CCT-Associated Indexes for Distinguishing Between PA and EH

#### CCT-Associated Single Indexes

**Figure 1:**
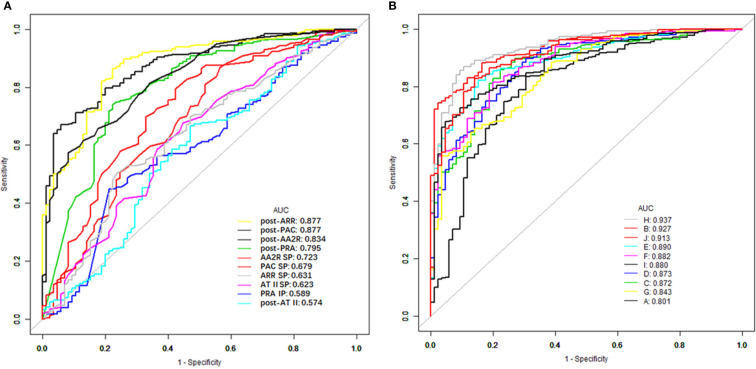
ROC curves of different CCT-associated single **(A)** and combined **(B)** indexes for distinguishing between PA and EH. ROC, receiver operating characteristic; CCT, captopril challenge test; PA, primary aldosteronism; EH, essential hypertension; AUC, area under the ROC curve; PAC, plasma aldosterone concentration; PRA, plasma renin activity; AT II, angiotensin II; ARR, aldosterone to renin ratio; AA2R, aldosterone to angiotensin II ratio; Post-PAC/PRA/AT II/ARR/AA2R, PAC/PRA/AT II/ARR/AA2R after the CCT; PAC/ATII/ARR/AA2R SP, suppression percentage of PAC/ATII/ARR/AA2R in the CCT; PRA IP, increasing percentage of PRA in the CCT; A, post-PRA + AA2R SP; B, post-PRA + post-PAC; C, post-PRA + post-ARR; D, post-PRA + post-AA2R; E, AA2R SP + post-PAC; F, AA2R SP + post-ARR; G, AA2R SP + post-AA2R; H, post-PAC + post-ARR; I, post-PAC + post-AA2R; J, post-ARR + post-AA2R.

The top 6 single indexes with the largest AUC for distinguishing PA from EH were post-ARR (0.8771, 95% CI 0.837–0.917), post-PAC (0.8769, 95% CI 0.839–0.915), post-AA2R (0.834, 95% CI 0.789–0.879), post-PRA (0.795, 95% CI 0.739–0.852), AA2R SP (0.723, 95% CI 0.660–0.787), and PAC SP (0.679, 95% CI 0.610–0.748), the AUC of which were all significantly larger than the area under the reference line (AUC = 0.5) (P<0.0001) (**Figure 1A** and **Table 3**). Pairwise comparison of the six single indexes showed significant differences between post-ARR and post-PRA (Z=7.226, P<0.0001), AA2R SP (Z=4.104, P<0.0001), or PAC SP (Z=5.051, P<0.0001); post-PAC and post-AA2R (Z=2.193, P=0.0283), post-PRA (Z=2.108, P = 0.0350), AA2R SP (Z=4.371, P<0.0001) or PAC SP (Z=5.284, P<0.0001); post-AA2R and AA2R SP (Z=3.637, P=0.0003), or PAC SP (Z=4.118, P<0.0001); post-PRA and PAC SP (Z=2.583, P=0.0098).

**Table 3:** The value of different CCT-associated single and combined indexes for distinguishing between PA and EH.

	AUC (95% CI)	Cutoff	Sen (95% CI) (%)	Spe (95% CI) (%)
Post-ARR ^*^	0.8771 (0.839–0.915)	23.0 (ng/dl)/(ng/ml/h)	86.5 (82.2–90.1)	77.7 (67.3–86.0)
Post-PAC ^*^	0.8769 (0.610–0.748)	20.2 ng/dl	67.0 (61.5–72.2)	94.1 (86.8–98.1)
Post-AA2R ^*^	0.834 (0.739–0.852)	0.4 (ng/dl)/(ng/L)	57.4 (51.7–62.9)	91.8 (83.8–96.6)
Post-PRA ^*^	0.795 (0.520–0.659)	0.6 ng/ml/h	74.0 (68.8–78.8)	77.7 (67.3–86.0)
AA2R SP ^*^	0.723 (0.502–0.647)	27.5%	79.5 (74.6–83.8)	57.7 (46.4–68.3)
PAC SP ^*^	0.679 (0.553–0.693)	30.5%	86.9 (82.6–90.4)	43.5 (32.8–54.7)
ARR SP ^*^	0.631 (0.837–0.917)	31.5%	50.3 (44.6–56.0)	76.5 (66.0–85.0)
AT II SP ^*^	0.623 (0.563–0.699)	−3.5%	67.6 (62.1–72.8)	55.3 (44.1–66.1)
PRA IP ^*^	0.589 (0.789–0.879)	1.0%	44.9 (39.3–50.6)	78.8 (68.6–86.9)
Post-AT II ^*^	0.574 (0.660–0.787)	61.4 ng/L	66.7 (61.1–71.9)	52.9 (41.8–63.9)
A (post-PRA + AA2R SP) ^*^	0.801 (0.745–0.858)	0.77	83.3 (78.7–87.3)	69.4 (58.5–79.0)
B (post-PRA + post-PAC) ^*^	0.927 (0.899–0.956)	0.87	74.4 (69.1–79.1)	97.7 (91.8–99.7)
C (post-PRA + post-ARR) ^*^	0.872 (0.832–0.913)	0.63	85.9 (81.5–89.6)	77.7 (67.3–86.0)
D (post-PRA+post-AA2R) ^*^	0.873 (0.831–0.914)	0.65	89.7 (85.8–92.9)	69.4 (58.5–79.0)
E (AA2R SP + post-PAC) ^*^	0.891 (0.855–0.927)	0.73	82.1 (77.3–86.1)	84.7 (75.3–91.6)
F (AA2R SP + post-ARR) ^*^	0.882 (0.845–0.919)	0.69	81.4 (76.6–85.6)	80.0 (69.9–87.9)
G (AA2R SP + post-AA2R) ^*^	0.843 (0.799–0.888)	0.89	55.8 (50.1–61.4)	96.5 (90.0–99.3)
H (post-PAC + post-ARR) ^*^	0.937 (0.911–0.963)	0.69	86.9 (82.6–90.4)	89.4 (80.8–95.0)
I (post-PAC + post-AA2R) ^*^	0.880 (0.844–0.917)	0.80	72.4 (67.1–77.3)	90.6 (82.3–95.8)
J (post-ARR+ post-AA2R)^*^	0.913 (0.882–0.944)	0.72	82.7 (78.0–86.7)	85.9 (76.6–92.5)

*Indicates that the AUC of the index is significantly larger than the area under the diagnostic reference line (P＜0.05). CCT, captopril challenge test; PA, primary aldosteronism; EH, essential hypertension; PAC, plasma aldosterone concentration; PRA, plasma renin activity; AT II, angiotensin II; ARR, aldosterone to renin ratio; AA2R, aldosterone to angiotensin II ratio; Post-PAC/PRA/AT II/ARR/AA2R, PAC/PRA/AT II/ARR/AA2R after the CCT; PAC/ATII/ARR/AA2R SP, suppression percentage of PAC/ATII/ARR/AA2R in the CCT; PRA IP, increasing percentage of PRA in the CCT; AUC, area under the receiver operating characteristic curve; Cutoff, the optimal cutoff value determined according to the Youden’s index; Sen, sensitivity; Spe, specificity; CI, confidence interval.

The AUC of PAC, AA2R or AT II was significantly larger after the CCT than that before the CCT (0.781, 95% CI 0.737–0.820 for pre-PAC; 0.729, 95% CI 0.682–0.772 for pre-AA2R; 0.508, 95% CI 0.524–0.623 for pre-AT II. P < 0.05), while the AUC of ARR and PRA did not differ significantly before and after the CCT (0.884, 95% CI 0.849–0.914 for pre-ARR; 0.811, 0.769–0.848 for pre-PRA. P > 0.05).

The optimal cutoff values for post-ARR, post-PAC, post-AA2R, post-PRA, AA2R SP, and PAC SP were 23.0 (ng/dl)/(ng/ml/h), 20.2 ng/dl, 0.4 (ng/dl)/(ng/L), 0.6 ng/ml/h, 27.5%, and 30.5%, respectively. The corresponding sensitivities for them were 86.5% (95% CI 82.2%–90.1%), 67.0% (95% CI 61.5%–72.2%), 57.4% (95% CI 51.7%–62.9%), 74.0% (95% CI 68.8%–78.8%), 79.5% (95% CI 74.6%–83.8%), and 86.9% (95% CI 82.6%–90.4%), respectively. The corresponding specificities for them were 77.7% (95% CI 67.3%–86.0%), 94.1% (95% CI 86.8%–98.1%), 91.8% (95% CI 83.8%–96.6%), 77.7% (95% CI 67.3%–86.0%), 57.7% (95% CI 46.4%–68.3%), and 43.5% (95% CI 32.8%–54.7%), respectively (**Table 3**).

#### Subgroup Analyses on the Value of CCT-Associated Single Indexes for Distinguishing Between PA and EH

Detailed information on the diagnostic value of single indexes in different subgroups is shown in **Table 4**.

**Table 4:** Subgroup analyses on the value of CCT-associated single indexes for distinguishing between PA and EH.

	AUC (95% CI)	Cutoff	Sen (95% CI) (%)	Spe (95% CI) (%)
PA subtype				
UPA subgroup (N=179)				
Post-PAC	0.914 (0.873–0.945)	20.9 ng/dl	78.2 (71.4–84.0)	96.5 (90.0–99.3)
Post-ARR	0.883 (0.838–0.919)	29.7 (ng/dl)/(ng/ml/h)	83.8 (77.6–88.9)	81.2 (71.2–88.8)
Post-AA2R	0.876 (0.830–0.913)	0.4 (ng/dl)/(ng/L)	70.4 (63.1–77.0)	91.8 (83.8–96.6)
Post-PRA	0.795 (0.741–0.842)	0.6 ng/ml/h	74.9 (67.8–81.0)	76.5 (66.0–85.0)
AA2R SP	0.712 (0.653–0.766)	27.0%	79.3 (72.7–85.0)	57.7 (46.4–68.3)
PAC SP	0.665 (0.605–0.722)	28.0%	82.1 (75.7–87.4)	48.2 (37.3–59.3)
BPA subgroup (N=97)				
Post-ARR	0.875 (0.818–0.919)	23.0 (ng/dl)/(ng/ml/h)	87.6 (79.4–93.4)	77.7 (67.3–86.0)
Post-PAC	0.827 (0.764–0.879)	19.4 ng/dl	61.9 (51.4–71.5)	89.4 (80.8–95.0)
Post-PRA	0.801 (0.736–0.856)	0.6 ng/ml/h	76.3 (66.6–84.3)	77.7 (67.3–86.0)
Post-AA2R	0.771 (0.703–0.830)	0.2 (ng/dl)/(ng/L)	92.8 (85.7–97.0)	47.1 (36.1–58.2)
AA2R SP	0.719 (0.648–0.783)	32.0%	86.6 (78.2–92.7)	49.4 (38.4–60.5)
PAC SP	0.694 (0.621–0.760)	17.0%	63.9 (53.5–73.4)	69.4 (58.5–79.0)
Posture during blood sampling				
“supine CCT” subgroup (N=101)				
Post-PAC	0.939 (0.892–0.986)	16.2 ng/dl	88.2 (78.1–94.8)	88.0 (68.8–97.5)
Post-ARR	0.876 (0.792–0.936)	29.7 (ng/dl)/(ng/ml/h)	83.8 (72.9–91.6)	88.0 (68.8–97.5)
Post-AA2R	0.793 (0.696–0.870)	0.5 (ng/dl)/(ng/L)	54.4 (41.9–66.5)	96.0 (79.6–99.9)
Post-PRA	0.772 (0.668–0.876)	0.6 ng/ml/h	75.0 (63.0–84.7)	80.0 (59.3–93.2)
“upright CCT” subgroup (N=296)				
Post-ARR	0.876 (0.828–0.925)	18.7 (ng/dl)/(ng/ml/h)	89.8 (85.2–93.4)	76.7 (64.0–86.6)
Post-AA2R	0.857 (0.807–0.907)	0.3 (ng/dl)/(ng/L)	75.0 (69.0–80.4)	80.0 (67.7–89.2)
Post-PAC	0.849 (0.799–0.898)	20.9 ng/dl	64.4 (57.9–70.5)	95.0 (86.1–99.0)
Post-PRA	0.804 (0.734–0.874)	1.4 ng/ml/h	91.1 (86.7–94.4)	66.7 (53.3–78.3)
AA2R SP	0.770 (0.703–0.837)	32.0%	86.4 (81.4–90.5)	58.3 (44.9–70.9)
PAC SP	0.728 (0.652–0.804)	28.0%	81.8 (76.3–86.5)	56.7 (43.2–69.4)
Serum potassium status				
“non-hypokalemia” subgroup (N=237)				
Post-ARR	0.873 (0.804–0.941)	19.4 (ng/dl)/(ng/ml/h)	90.0 (79.5–96.2)	71.7 (56.5–84.0)
Post-PRA	0.815 (0.730–0.899)	0.4 ng/ml/h	78.3 (65.8–87.9)	76.1 (61.2–87.4)
Post-PAC	0.756 (0.663–0.849)	15.5 ng/dl	78.3 (65.8–87.9)	69.6 (54.2–82.3)
AA2R SP	0.742 (0.646–0.838)	27.0%	80.0 (67.7–89.2)	63.0 (47.5–76.8)
PAC SP	0.728 (0.630–0.827)	17.0%	63.3 (49.9–75.4)	76.1 (61.2–87.4)
Post-AA2R	0.709 (0.609–0.809)	0.3 (ng/dl)/(ng/L)	65.0 (51.6–76.9)	69.6 (54.2–82.3)
“hypokalemia” subgroup (N=160)				
Post-PAC	0.895 (0.842–0.947)	20.1 ng/dl	76.2 (70.4–81.3)	92.3 (79.1–98.4)
Post-ARR	0.887 (0.833–0.940)	23.0 (ng/dl)/(ng/ml/h)	86.5 (81.7–90.5)	82.1 (66.5–92.5)
Post-AA2R	0.849 (0.786–0.912)	0.4 (ng/dl)/(ng/L)	69.8 (63.8–75.4)	84.6 (69.5–94.1)
Post-PRA	0.810 (0.732–0.887)	0.6 ng/ml/h	73.8 (67.9–79.1)	84.6 (69.5–94.1)
AA2R SP	0.699 (0.607–0.792)	22.0%	71.4 (65.4–76.9)	64.1 (47.2–78.8)
ARR SP	0.693 (0.602–0.784)	43.0%	59.9 (53.6–66.0)	76.9 (60.7–88.9)
Finding on adrenal CT				
“normal imaging” subgroup (N=100)				
Post-PAC	0.864 (0.767–0.960)	16.8 ng/dl	78.6 (63.2–89.)	84.6 (65.1–95.6)
Post-ARR	0.858 (0.763–0.954)	29.7 (ng/dl)/(ng/ml/h)	76.2 (60.5–87.9)	84.6 (65.1–95.6)
Post-PRA	0.792 (0.673–0.911)	0.6 ng/ml/h	78.6 (63.2–89.7)	76.9 (56.4–91.0)
Post-AA2R	0.721 (0.593–0.849)	0.2 (ng/dl)/(ng/L)	88.1 (74.4–96.0)	46.2 (26.6–66.6)
AA2R SP	0.708 (0.577–0.840)	15.0%	64.3 (48.0–78.4)	76.9 (56.4–91.0)
PAC SP	0.702 (0.566–0.839)	31.0%	92.9 (80.5–98.5)	50.0 (29.9–70.1)
“abnormal imaging” subgroup (N=297)				
Post-PAC	0.885 (0.844–0.926)	20.5 ng/dl	68.6 (62.6–74.2)	96.2 (86.8–99.5)
Post-ARR	0.875 (0.826–0.925)	21.4 (ng/dl)/(ng/ml/h)	88.1 (83.6–91.8)	76.9 (63.2–87.5)
Post-AA2R	0.860 (0.811–0.909)	0.3 (ng/dl)/(ng/L)	67.4 (61.4–73.1)	88.5 (76.6–95.6)
Post-PRA	0.795 (0.724–0.866)	0.7 ng/ml/h	77.8 (72.2–82.7)	76.9 (63.2–87.5)
AA2R SP	0.741 (0.665–0.818)	27.0%	78.9 (73.5–83.7)	61.5 (47.0–74.7)
PAC SP	0.669 (0.582–0.755)	18.0%	58.2 (52.0–64.3)	69.2 (54.9–81.3)

The AUC of all the indexes shown in the table are significantly larger than the area under the diagnostic reference line (AUC=0.5) (P＜0.05). For a subgroup with more than six indexes whose AUC are significantly larger than 0.5, only the top 6 single indexes with the largest AUC are shown. CCT, captopril challenge test; PA, primary aldosteronism; EH, essential hypertension; UPA, unilateral primary aldosteronism; BPA, bilateral primary aldosteronism; “supine CCT”, the CCT conducted in the supine position; “upright CCT”, the CCT conducted in the upright position; “non-hypokalemia”, the CCT conducted in patients without hypokalemia; “hypokalemia”, the CCT conducted in patients with hypokalemia; “normal imaging”, the CCT conducted in patients with normal findings on adrenal CT; “abnormal imaging”, the CCT conducted in patients with abnormal findings on adrenal CT; PAC, plasma aldosterone concentration; PRA, plasma renin activity; AT II, angiotensin II; ARR, aldosterone to renin ratio; AA2R, aldosterone to angiotensin II ratio; Post-PAC/PRA/AT II/ARR/AA2R, PAC/PRA/AT II/ARR/AA2R after the CCT; PAC/ATII/ARR/AA2R SP, suppression percentage of PAC/ATII/ARR/AA2R in the CCT; PRA IP, increasing percentage of PRA in the CCT; AUC, area under the receiver operating characteristic curve; Cutoff, the optimal cutoff value determined according to the Youden’s index; Sen, sensitivity; Spe, specificity; CI, confidence interval.

#### Subgroup Analysis by the PA Subtype (UPA or BPA)

For distinguishing UPA from EH (UPA subgroup), the top 6 single indexes with the largest and significant AUC were post-PAC, post-ARR, post-AA2R, post-PRA, AA2R SP, and PAC SP. Their AUC from largest to smallest could be ordered as follows: post-PAC (0.914) > post-AA2R (0.876) > post-PRA (0.795), AA2R SP (0.712) or PAC SP (0.665); post-ARR (0.883) > post-PRA, AA2R SP or PAC SP; post-PRA > PAC SP (P < 0.05).

For distinguishing BPA from EH (BPA subgroup), the top 6 single indexes with the largest and significant AUC were post-ARR, post-PAC, post-PRA, post-AA2R, AA2R SP, and PAC SP. Their AUC from largest to smallest could be ordered as follows: post-ARR (0.875) > post-PRA (0.801), post-AA2R (0.771), AA2R SP (0.719) or PAC SP (0.694); post-PAC (0.827) > AA2R SP, or PAC SP; post-PRA > PAC SP (P < 0.05).

The AUC of post-ARR was not significantly different in the two subgroups (P>0.05), while the AUC of post-PAC was significantly higher in the UPA than BPA subgroup (P<0.05) (**Table 4**).

#### Subgroup Analysis by the Posture (Supine or Upright Position) During Blood Sampling

For the CCT in the supine position (“supine CCT” subgroup), the top 6 single indexes with the largest and significant AUC for distinguishing PA from EH were post-PAC, post-ARR, post-AA2R, and post-PRA. Their AUC from largest to smallest could be ordered as follows: post-PAC (0.939) > post-AA2R (0.793) or post-PRA (0.772); post-ARR (0.876) > post-PRA (P < 0.05).

For the CCT in the upright position (“upright CCT” subgroup), the top 6 single indexes with the largest and significant AUC for distinguishing PA from EH were post-ARR, post-AA2R, post-PAC, post-PRA, AA2R SP, and PAC SP. Their AUC from largest to smallest could be ordered as follows: post-ARR (0.876) > post-PRA (0.804), AA2R SP (0.770) or PAC SP (0.728); post-AA2R (0.857) or post-PAC (0.849) > AA2R SP or PAC SP (P < 0.05).

The AUC of post-ARR was not significantly different in the two subgroups (P>0.05), while the AUC of post-PAC was significantly higher in the “supine CCT” than “upright CCT” subgroup (P<0.05) (**Table 4**).

#### Subgroup Analysis by the Serum Potassium Status (Without or With Hypokalemia)

For the CCT conducted in patients without hypokalemia (“non-hypokalemia” subgroup), the top 6 single indexes with the largest and significant AUC for distinguishing PA from EH were post-ARR, post-PRA, post-PAC, AA2R SP, PAC SP, and post-AA2R. Their AUC from largest to smallest could be ordered as follows: post-ARR (0.873) > post-PRA (0.815), AA2R SP (0.742), PAC SP (0.728) or post-AA2R (0.709) (P < 0.05). Post-PAC (AUC=0.756) was not ordered with the other five indexes because it did not have a significantly different AUC from the others (P > 0.05).

For the CCT conducted in patients with hypokalemia (“hypokalemia” subgroup), the top 6 single indexes with the largest and significant AUC for distinguishing PA from EH were post-PAC, post-ARR, post-AA2R, post-PRA, AA2R SP, and ARR SP. Their AUC from largest to smallest could be ordered as follows: post-PAC (0.895) > AA2R SP (0.699) or ARR SP (0.693); post-ARR (0.887) > post-PRA (0.810), AA2R SP or ARR SP; post-AA2R (0.849) > AA2R SP or ARR SP; post-PRA > ARR SP (P < 0.05).

The AUC of post-ARR was not significantly different in the two subgroups (P>0.05), while the AUC of post-PAC was significantly higher in the “hypokalemia” than “non-hypokalemia” subgroup (P<0.05) (**Table 4**).

#### Subgroup Analysis by the Finding on Adrenal CT (Normal or Abnormal Imaging)

For the CCT conducted in patients with normal findings on adrenal CT (“normal imaging” subgroup), the top 6 single indexes with the largest and significant AUC for distinguishing PA from EH were post-PAC, post-ARR, post-PRA, post-AA2R, AA2R SP, and PAC SP. Their AUC from largest to smallest could be ordered as follows: post-PAC (0.864) > post-AA2R (0.721), AA2R SP (0.708) or PAC SP (0.702); post-ARR (0.858)> post-PRA (0.792), or PAC SP (P < 0.05).

For the CCT conducted in patients with abnormal findings on adrenal CT (“abnormal” subgroup), the top 6 single indexes with the largest and significant AUC for distinguishing PA from EH were post-PAC, post-ARR, post-AA2R, post-PRA, AA2R SP, and PAC SP. Their AUC from largest to smallest could be ordered as follows: post-PAC (0.885) or post-ARR (0.875) > post-PRA (0.795), AA2R SP (0.741) or PAC SP (0.669); post-AA2R (0.860) > AA2R SP > PAC SP; post-PRA > PAC SP (P < 0.05).

The AUCs of post-ARR and post-PAC were neither significantly different in the two subgroups (P>0.05) (**Table 4**).

#### CCT-Associated Combined Indexes

We combined any two of the top 5 singles indexes with the largest AUC and thus formed ten new combined indexes represented by A-J as showed in **Table 3** (“+” means “combined with”). For assessment on their diagnostic value, binary logistic regression models in which the corresponding variables entered were developed, and predicted probabilities of distinguishing PA from EH were calculated using formulas in **Table 5**. For example, probabilities using the combined index “post-PRA + AA2R SP” were calculated using the following formula: ŷ = 1/[1 + exp. (−2.265 + 0.217 × post-PRA + 3.063 × AA2R SP)].

**Table 5:** Formulas of calculating predicted probabilities of distinguishing PA from EH using different combined indexes.

Combined index	Formula
A (post-PRA + AA2R SP)	ŷ = 1/[1 + exp. (−2.265 + 0.217 × post-PRA + 3.063 × AA2R SP)]
B (post-PRA + post-PAC)	ŷ = 1/[1 + exp. (4.330 + 0.346 × post-PRA − 0.345 × post-PAC)]
C (post-PRA + post-ARR)	ŷ = 1/[1 + exp. (−0.03 + 0.061 × post-PRA − 0.024 × post-ARR)]
D (post-PRA+post-AA2R)	ŷ = 1/[1 + exp. (1.604 + 0.224 × post-PRA − 9.879 × post-AA2R)]
E (AA2R SP + post-PAC)	ŷ = 1/[1 + exp. (3.167 + 2.282 × AA2R SP − 0.259 × post-PAC)]
F (AA2R SP + post-ARR)	ŷ = 1/[1 + exp. (−0.353 + 2.945 × AA2R SP − 0.026 × post-ARR)]
G (AA2R SP + post-AA2R)	ŷ = 1/[1 + exp. (1.356 + 1.702 × AA2R SP − 8.673 × post-AA2R)]
H (post-PAC + post-ARR)	ŷ = 1/[1 + exp. (5.250–0.271 × post-PAC − 0.029 × post-ARR)]
I (post-PAC + post-AA2R)	ŷ = 1/[1 + exp. (3.999– 0.240 × post-PAC − 2.340 × post-AA2R)]
J (post-ARR+ post-AA2R)	ŷ = 1/[1 + exp. (3.141– 0.026 × post-ARR − 9.089 × post-AA2R)]

ŷ, predicted probabilities of distinguishing PA from EH.

Then the AUC of each combined index was computed based on the corresponding predicted probabilities of patients. Among these combined indexes, the top 6 ones with the largest AUC for distinguishing PA from EH were “H (post-PAC + post-ARR)” (0.937, 95% CI 0.911–0.963), “B (post-PRA + post-PAC)” (0.927, 95% CI 0.899–0.956), “J (post-ARR+ post-AA2R)” (0.913, 95% CI 0.882–0.944), “E (AA2R SP + post-PAC)” (0.891, 95% CI 0.855–0.926), “F (AA2R SP + post-ARR)” (0.882, 95% CI 0.845–0.919), and “I (post-PAC + post-AA2R)” (0.880, 95% CI 0.844–0.917), the AUC of which were all significantly larger than the area under the reference line (AUC=0.5) (P>0.0001) (**Figure 1B**, **Table 3**). Pairwise comparison of the six combined indexes showed significant differences between “H (post-PAC + post-ARR)” and “J (post-ARR+ post-AA2R)” (Z=2.557, P=0.0106), “E (AA2R SP + post-PAC)” (Z=3.159, P=0.0016), “F (AA2R SP + post-ARR)” (Z=3.406, P=0.0007), or “I (post-PAC + post-AA2R)” (Z=3.899, P=0.0001); “B (post-PRA + post-PAC)” and “E (AA2R SP + post-PAC)” (Z=2.859, P=0.0043), “F (AA2R SP + post-ARR)” (Z=2.236, P=0.0253) or “I (post-PAC + post-AA2R)” (Z=3.583, P=0.0003); “J (post-ARR+ post-AA2R)” and “F (AA2R SP + post-ARR)” (Z=2.299, P=0.0215).

“H (post-PAC + post-ARR)” had a larger AUC than “pre-PAC + pre-ARR” (0.906, 95% CI 0.873–0.933) (Z=2.446, P = 0.0145), while the AUC of “B (post-PRA + post-PAC)” or “J (post-ARR+ post-AA2R)” was not significantly larger than that of “pre-PRA + pre-PAC” (0.908, 95% CI 0.875–0.935) or “pre-ARR + pre-AA2R” (0.891, 95% CI 0.857–0.920), respectively (P > 0.05).

The optimal cutoff values of combined indexes shown in **Table 3** were actually predicted probabilities obtained by calculation as described above. For example, “H (post-PAC + post-ARR)” had an optimal threshold at 0.69, meaning that if patients whose post-PAC and post-ARR values resulted in a predicted probability over 69% according to its logistic equation were identified to have PA, this combined index could achieve its highest diagnostic efficacy. Then at the corresponding cutoff probabilities, the sensitivities for “H (post-PAC + post-ARR)”, “B (post-PRA + post-PAC)”, “J (post-ARR+ post-AA2R)”, “E (AA2R SP + post-PAC)”, “F (AA2R SP + post-ARR)” and “I (post-PAC + post-AA2R)” were 86.9% (95% CI 82.6%–90.4%), 74.4% (95% CI 69.1%–79.1%), 82.7% (95% CI 78.0%–86.7%), 82.1% (95% CI 77.3%–86.1%), 81.4% (95% CI 76.6%–85.6%), and 72.4% (95% CI 67.1%–77.3%), respectively. The corresponding specificities for them were 89.4% (95% CI 80.8%–95.0%), 97.7% (95% CI 91.8%–99.7%), 85.9% (95% CI 76.6%–92.5%), 84.7% (95% CI 75.3%–91.6%), 80.0% (95% CI 69.9%–87.9%), and 90.6% (95% CI 82.3%–95.8%), respectively (**Table 3**).

Pairwise comparison between the single and combined indexes showed that post-ARR had a significantly lower AUC than “B (post-PRA + post-PAC)” (Z=2.282, P=0.0225), “H (post-PAC + post-ARR)” (Z=3.407, P=0.0007), or “J (post-ARR+ post-AA2R)” (Z=2.267, P=0.0234); post-PAC had a significantly lower AUC than “B (post-PRA + post-PAC)” (Z=3.590, P=0.0003) or “H (post-PAC + post-ARR)” (Z=3.976, P=0.0001).

## Discussion

Based on the EMR system of our hospital, we retrospectively evaluated the value of different CCT-associated indexes in the diagnosis of PA. We tried to include all the single indexes which were recommended by the guidelines or consensus, used for result interpretation in clinical practice, or rarely investigated in studies. We also assessed different combinations of the single indexes, which had not been greatly valued before. To the best of our knowledge, this study provides us the most comprehensive understanding about the diagnostic value of various CCT-associated indexes.

The rates of hypokalemia (22.35%) and abnormal findings on adrenal CT (32.94%) in EH were not low in our study, although the rates were significantly lower than those in PA. Even for patients with BPA, 73.20% of them had adrenal lesions on CT, although the proportion was higher for patients with UPA. The explanation may be that many patients with hypertension along with an incidentally found adrenal mass were evaluated for suspicion of PA, and although a large portion of them were eventually confirmed to have EH with a non-functional adrenal incidentaloma, some of them still underwent adrenalectomy due to consideration about the tumor size or site. A possible reason for hypokalemia in EH is that some forms of EH are also associated with low renin levels, and hypokalemia can be an important finding in this form of EH (31).

In terms of distinguishing between PA and EH, our study showed that post-ARR and post-PAC had comparably high diagnostic efficacy among all the single indexes. The Japanese guidelines (11) recommended that patients with post-PAC/PRA>200 (pg/ml)/(ng/ml/h) or 20 (ng/dl)/(ng/ml/h) should be confirmed to have PA. Previous studies on post-ARR have reported sensitivities of 59% to 100% and specificities of 82.76% to 99% at optimal cutoff values of 20 to 35 (ng/dl)/(ng/ml/h) (7, 13, 15–23, 32, 33), while our study showed a similar optimal cutoff value (23.0 (ng/dl)/(ng/ml/h)) and sensitivity (86.5%) but a relatively lower specificity (77.7%). However, post-ARR was not significantly superior to pre-ARR in our study. The Japanese guidelines (11) also recommended that the CCT is judged to be positive with post-PAC >120 pg/ml. Our study showed high diagnostic efficacy of post-PAC which was even superior to that of pre-PAC. However, results of studies on post-PAC are controversial in terms of its diagnostic value and optimal cutoff values (11, 13, 14), but are generally consistent with our results. Our finding was also supported by the prospective diagnostic accuracy study by Song Y et al. (34) showing that post-PAC was highly recommended for interpreting CCT results. The rarely studied index, post-AA2R, whose diagnostic efficacy was higher than that of pre-AA2R, showed a high specificity (91.8%) but poor sensitivity (57.4%) at an optimal cutoff value of 0.4 (ng/dl)/(ng/L) in our study. Surprisingly, PAC SP, which is currently recommended in guidelines (2, 12) and most widely used in clinical practice with post-CCT PAC not suppressed or suppressed less than 30% as the judgment criterion, showed low diagnostic efficacy in our study which was even lower than post-ARR, post-PAC, post-AA2R, and post-PRA. Although aldosterone can be suppressed by over 30% in the CCT of normal people (2, 12), it does not necessarily mean that patients with PAC SP<30% should be confirmed to have PA. In our study, we also noticed that although aldosterone was more greatly suppressed in the EH (by 26%) than PA group (by 14%), which might be caused by stronger autonomous secretion of aldosterone in PA than EH, the suppression percentages in both groups were less than 30%, which was the cutoff value recommended in guidelines (2, 12). This is consistent with findings in several Chinese studies that PAC SP of EH patients ranged from 2% to 10%, which was similar to that of PA patients ranging from 0.8% to 17.4% (25–27). Therefore, it may be difficult to distinguish between PA from low-renin essential hypertension, and caution should be taken to interpret CCT results with PAC SP due to the lack of sufficient data on changes in aldosterone levels of EH patients. The reason remains unclear, but may be related to high dietary salt intake of Chinese people, which can suppress the activity of the RAAS and thus lead to a low response to captopril (35). However, post-PRA showed moderately high diagnostic efficacy in our study, which was lower than post-ARR, post-PAC but higher than PAC SP. More evidence is expected to support the value of post-PRA in differentiating PA from EH. The combination of PAC and ARR after the CCT achieved higher diagnostic efficacy than either alone, which was even superior to their combination before the CCT. Therefore, we can take post-ARR and post-PAC altogether into account in clinical practice to aid in diagnosis of PA. In addition, different indexes can be chosen when different diagnostic aspects are of greater importance, such as reducing misdiagnoses or missed diagnoses.

Subgroup analyses showed that the PA subtype, posture during blood sampling in the CCT and serum potassium status might not influence the diagnostic value of post-ARR, but post-PAC might perform better in the CCT in the supine position or conducted in patients with hypokalemia. However, two studies by Stowasser M et al. (36) and Ahmed AH et al. (37) revealed that seated SIT was highly sensitive and superior to recumbent SIT in identifying PA, and a Chinese study (27) showed that seated CCT could replace recumbent CCT as a more confirmatory test, which could be explained by the activation of RAAS in the upright position in EH patients but autonomous, dysfunctional secretion of aldosterone in PA patients. The reason why our finding argued against this hypothesis remains unclear but may be associated with the fact that only a small portion of patients (68 PA patients and 25 EH patients) in our study conducted the CCT in the supine position, which could possibly lead to incorrect estimates. Whether patients had abnormal findings on adrenal CT or not did not make a difference in the diagnostic value of post-ARR and post-PAC. It was noteworthy that post-PAC was shown to perform better in distinguishing the unilateral than bilateral form of PA from EH, and the underlying mechanism may be that mineralocorticoid excess is more severe in UPA than BPA. This was consistent with the view that response of aldosterone after the CCT may differ between APA and IHA. Although the subtype classification of PA based on the pathologic diagnosis (APA or IHA) does not completely equal to that based on the lateralized status (UPA or BPA), APA or IHA, respectively, makes up a majority of the lateralized (unilateral) or non-lateralized (bilateral) form of PA (1–3, 38).

In this study, we developed unified criteria for inclusion and exclusion for PA, EH, UPA and BPA, and we tried to make sure that all the patients followed standard procedures according to recommendations, such as discontinuation of specific types of medications and periods of withdrawal. We chose the latest screening test during hospitalization to avoid potential interference with testing results, and among various records of serum potassium of each patient, we chose the value in the blood biochemical test concomitant with the CCT for analyses, because potassium level is one of the major determinants for aldosterone secretion. Radioimmunoassay, which was commonly recommended by guidelines (2, 11, 12), was adopted for the measurement of PAC, PRA and AT II. This standardization helped to assure the validity of our findings. Besides, no significant differences in gender, age, BMI, SBP, and eGFR between patients with PA and EH ensured comparable demographic and medical baseline characteristics to some extent. However, age and BMI significantly differed between UPA and BPA, posing a potential bias to the results in these subgroups, which needed to be better addressed in future research to avoid interference.

However, there were some limitations in our study. First, the retrospective nature of the study with the long enrolment might introduce a potential selection bias. It is important to note that the percentage of hypokalemia in PA patients in our study was rather high compared with previous studies, which might point to more severe PA in the patients included in this study. Therefore, our study population might not be representative enough of PA patients with differing characteristics and milder forms, but to some extent, our findings may still be meaningful for clinical settings where the form of PA in patients admitted to hospital for confirmation tends to be more severe, and we expect more evidence from prospective clinical studies with good designs and large sample sizes to verify our conclusions, in which patients with a broader range of disease severity can be included. Second, as described before, the rates of abnormal findings on adrenal CT in EH, BPA and hypokalemia in EH were not low, which could be different from those of other centers (5, 39, 40) and limit generalization of our findings. Third, noncosyntropin-stimulated AVS was performed with sequential cannulation, which would result in inappropriate lateralization in a minority of cases (41). Fourth, we could not identify subtypes of PA from the perspective of the pathologic diagnosis (APA or IHA) due to insufficient post-operative pathological evidence by immunohistochemical staining and follow-up data. However, as stated above, APA or IHA respectively overlaps a lot with the lateralized (unilateral) or non-lateralized (bilateral) form of PA (1–3, 38); more importantly, our subtype classification of PA patients based on the lateralized status was of great significance for selecting the most appropriate treatment, namely, adrenalectomy for the unilateral subtype or mineralocorticoid receptor antagonists for the bilateral subtype (42). Fifth, all the enrolled patients were Chinese, and considering ethnic, regional or dietary differences, disparity may exist between our results and evidence from studies conducted in the western population. For example, the optimal cutoff values for the post-ARR in Chinese studies are generally higher than those in studies performed in western countries (7, 13, 15–23, 32, 33), which may be caused by a lower response to captopril in the Chinese population due to long-term high dietary salt intake (35), although another Chinese study revealed no significant effect of dietary sodium intake on serum aldosterone levels (43). Further studies are needed to clarify the exact mechanism, and we should interpret CCT results based on various factors, such as patients’ clinical characteristics, sample size, ethic and regional features.

In conclusion, our study suggests that we can take post-CCT ARR and PAC altogether into account to distinguish PA from EH, while caution should be taken to interpret CCT results with the suppression percentage of PAC. The diagnostic value of post-CCT ARR may not be influenced by the PA subtype, posture during blood sampling in the CCT, serum potassium status, and finding on adrenal CT. However, post-CCT PAC may have higher diagnostic efficacy in the CCT in the supine position or conducted in patients with hypokalemia, and it may also perform better to identify the unilateral than bilateral form of PA. More evidence from prospective clinical studies with good designs and large sample sizes is expected to result in a more robust conclusion.

## Data Availability Statement

The original contributions presented in the study are included in the article/supplementary material. Further inquiries can be directed to the corresponding authors.

## Ethics Statement

The studies involving human participants were reviewed and approved by the Ethical Committee of Sichuan University West China Hospital. Written informed consent for participation was not required for this study in accordance with the national legislation and the institutional requirements.

## Author Contributions

YR and QX designed the project. QX, KY, and YL collected the data, reviewed the data, and checked the data with QL and TC. QX analyzed the data, wrote the manuscript. HT and YR supervised the project, reviewed, and revised the manuscript. All authors contributed to the article and approved the submitted version.

## Funding

This study was supported by the following grants: 1.3.5 Project for Disciplines of Excellence, West China Hospital, Sichuan University, grant ZYGD18022; 1·3·5 Project for Disciplines of Excellence–Clinical Research Incubation Project, West China Hospital, Sichuan University, grant 2018HXFH009; Sichuan Science and Technology Program-Applied Basic Research Project, grant 2019YJ0040.

## Conflict of Interest

The authors declare that the research was conducted in the absence of any commercial or financial relationships that could be construed as a potential conflict of interest.

## Abbreviations

PA, primary aldosteronism; EH, essential hypertension; APA, aldosterone-producing adenoma; IHA, idiopathic hyperaldosteronism; UPA, unilateral primary aldosteronism; BPA, bilateral primary aldosteronism; CCT, captopril challenge test; BMI, body mass index; eGFR, estimated glomerular filtration; SBP, systolic blood pressure; DBP, diastolic blood pressure; PAC, plasma aldosterone concentration; PRA, plasma renin activity; AT II, angiotensin II; ARR, aldosterone to renin ratio; AA2R, aldosterone to angiotensin II ratio; ROC curve, receiver operating characteristic curve; AUC, area under the receiver operating characteristic curve; Sen, sensitivity; Spe, specificity; CI, confidence interval; pre-PAC/PRA/AT II/ARR/AA2R, PAC/PRA/AT II/ARR/AA2R before the CCT; post-PAC/PRA/ATII/ARR/AA2R, PAC/PRA/AT II/ARR/AA2R after the CCT; PAC/ATII/ARR/AA2R SP, suppression percentage of PAC/ATII/ARR/AA2R in the CCT; PRA IP, increasing percentage of PRA in the CCT.
